# Comparative genomics allowed the identification of drug targets against human fungal pathogens

**DOI:** 10.1186/1471-2164-12-75

**Published:** 2011-01-27

**Authors:** Ana Karina R Abadio, Erika S Kioshima, Marcus M Teixeira, Natalia F Martins, Bernard Maigret, Maria Sueli S Felipe

**Affiliations:** 1 Department of Cellular Biology, University of Brasília, Brasília, Brazil; 2 Embrapa - Genetic Resources and Biotechnology, Brasília, Brazil; 3 Lorrain Laboratory of Computing Research and its Applications, University Henri Poincaré-Nancy I, Nancy, France

## Abstract

**Background:**

The prevalence of invasive fungal infections (IFIs) has increased steadily worldwide in the last few decades. Particularly, there has been a global rise in the number of infections among immunosuppressed people. These patients present severe clinical forms of the infections, which are commonly fatal, and they are more susceptible to opportunistic fungal infections than non-immunocompromised people. IFIs have historically been associated with high morbidity and mortality, partly because of the limitations of available antifungal therapies, including side effects, toxicities, drug interactions and antifungal resistance. Thus, the search for alternative therapies and/or the development of more specific drugs is a challenge that needs to be met. Genomics has created new ways of examining genes, which open new strategies for drug development and control of human diseases.

**Results:**

*In silico *analyses and manual mining selected initially 57 potential drug targets, based on 55 genes experimentally confirmed as essential for *Candida albicans *or *Aspergillus fumigatus *and other 2 genes (*kre2 *and *erg6*) relevant for fungal survival within the host. Orthologs for those 57 potential targets were also identified in eight human fungal pathogens (*C. albicans*, *A. fumigatus*, *Blastomyces dermatitidis*, *Paracoccidioides brasiliensis*, *Paracoccidioides lutzii, Coccidioides immitis*, *Cryptococcus neoformans *and *Histoplasma capsulatum*). Of those, 10 genes were present in all pathogenic fungi analyzed and absent in the human genome. We focused on four candidates: *trr1 *that encodes for thioredoxin reductase, *rim8 *that encodes for a protein involved in the proteolytic activation of a transcriptional factor in response to alkaline pH, *kre2 *that encodes for α-1,2-mannosyltransferase and *erg6 *that encodes for Δ(24)-sterol C-methyltransferase.

**Conclusions:**

Our data show that the comparative genomics analysis of eight fungal pathogens enabled the identification of four new potential drug targets. The preferred profile for fungal targets includes proteins conserved among fungi, but absent in the human genome. These characteristics potentially minimize toxic side effects exerted by pharmacological inhibition of the cellular targets. From this first step of post-genomic analysis, we obtained information relevant to future new drug development.

## Background

The frequency and diversity of invasive fungal infections have changed over the last 25 years. The emergence of less common, but medically important, fungi has increased, especially in the large populations of immunocompromised patients and of those hospitalized with serious underlying diseases [1,2]. These patients develop more severe clinical forms of mycoses, which are commonly fatal, and they are more susceptible to infections by opportunistic fungi than non-immunocompromised people [3]. The antifungal agents currently available for the treatment of systemic mycoses include four groups of drugs: polyenes (amphotericin B), azoles (fluconazole, itraconazole, ketoconazole, posaconazole and voriconazole), echinocandins (caspofungin, anidulafungin, and micafungin) and flucytosines [4]. Conventional amphotericin B, despite being a broad-spectrum fungicidal agent with little intrinsic or acquired resistance, is limited by its serious toxicities and lack of an oral formulation for systemic therapy. In recent years, three lipid formulations of amphotericin B (amphotericin B lipid complex, amphotericin B cholesteryl sulfate and liposomal amphotericin B) have been developed and approved by the Food and Drug Administration (FDA). Although less nephrotoxic than deoxycholate amphotericin B, lipid amphotericin B nephrotoxicity still limits treatment compared to the newer triazoles and echinocandins [5]. The triazoles are the most widely used antifungal agents and have activity against many fungal pathogens, with less serious nephrotoxic effects observed than with amphotericin B. However, the azoles antifungals have many drug-drug interactions with multiple drug classes owing to their interference with hepatic cytochrome P-450 enzymes [6]. Another problem with azoles therapy is the acquired resistance of many pathogens to these drugs, which is the most common cause of refractory infection. Thus, the search for alternative therapies and/or the development of more specific drugs is a challenge. Recently, efforts have been devoted to the chemistry side of discovering new antifungal agents, including the development of third-generation azoles or a new therapeutic class of antifungal drugs, such as echinocandins [7]. Additionally, nanotechnology approaches have improved the development of innovative products that reduce side effects by lowering dose administration of already available drugs, such as amphotericin B nanoencapsulated [8-10]. Many advances have been made in antifungal drug development in the past decade. However, the search for more specific drugs, in an effort to overcome the global problem of resistance to antifungal agents and minimize the serious side effects, is increasingly relevant and necessary.

Currently, drug research and development are expensive and time consuming. An estimated 14 years and an average of $1.8 billion is the investment required to develop a new drug that will reach the market [11]. Selecting new molecular targets by comparative genomics, homology modeling and virtual screening of compounds is promising in the process of new drug discovery. In fact, technological advances over the past two decades have led to the accumulation of genome-wide sequence data for many different fungal species. As the number of sequenced genomes rapidly increases, searching and comparing sequence features within and between species has become a part of most biological inquires [12]. Currently, 183 fungi genomes have been sequenced, either completely or are in the process of sequencing, and 40 human pathogenic fungi genomes have been sequenced. (Data collected on 09/07/2010 in the following databases: Fungal Genomes, TIGR, Sanger, Broad Institute and NCBI). Seven of the human pathogens are of great importance in systemic mycosis: *Candida albicans*, *Aspergillus fumigatus*, *Blastomyces dermatitidis*, *Coccidioides immitis*, *Cryptococcus neoformans*, *Paracoccidioides brasiliensis *and *Histoplasma capsulatum*, which are strong candidates for post-genomic studies.

Comparative genomics strategy is a useful tool in identifying potential new drug targets, such as putative essential genes and/or those affecting the cell viability that are conserved in pathogenic organisms [13-16]. By this methodology, ten genes conserved in three bacteria species (*Staphylococcus aureus*, *Mycobacterium tuberculosis *and *Escherichia coli *0157: H7) were selected as candidates for an antibacterial drug [14]. Since the publication of the nematode *Brugia malayi *complete genome, Kumar and colleagues [15] conducted a comparison analysis between the genomes of *B. malayi *and *Caenorhabditis elegans *and were able to identify 7,435 orthologs genes, from which 589 were identified as essential, as well as absent in the human genome, resulting in a list of candidate target genes for new drug development. Recently, Caffrey and colleagues [16] identified new drug targets in the metazoan pathogen *Schistosoma mansoni*, the causative agent of Schistosomiasis. The authors identified 35 orthologs essential genes and potential drug targets against this human pathogen.

Here we identified potential drug targets applied to human fungal pathogens using comparative genomics strategy. Ten genes were present in all pathogenic fungi analyzed and absent in the human genome. Among them, four genes (*trr1*, *rim8*, *kre2 *and *erg6*) were selected for future research and new drug development. Two of those genes codify for proteins (TRR1 and KRE2) that showed significant identity when compared to templates already deposited in the databank PDB (Protein Database Bank), which were used to perform homology modeling of both enzymes. These results will be used to virtually screen combinatorial libraries, offering new perspectives on technological development and innovation of antifungal agents against human pathogens.

## Results and Discussion

### Drug target selection

Direct demonstration of *A. fumigatus *and *C. albicans *gene essentiality was achieved using conditional promoter replacement (CPR) [17] and gene replacement and conditional expression (GRACE) strategies [18], respectively. Therefore, the initial ensemble of genes, experimentally described as essential in *C. albicans *and/or *A. fumigatus *were used to identify 55 orthologs. In addition, two non-essential genes (*kre2 *and *erg6*), but which are important to cell viability within the host [19,20], were added to the list of possible drug targets. The alignments of those 57 sequences against the genome of the 8 pathogenic fungi *P. lutzii*, *P. brasiliensis *isolates (Pb18 e Pb3), *A. fumigatus*, *B. dermatitidis*, *C. albicans*, *C. immitis*, *C. neoformans*, *H. capsulatum *confirmed the presence of all the genes (Additional file 1). As a result, ten conserved genes were selected as drug targets because they were present in all species analyzed and were also absent in the human genome, as shown in Table 1.

**Table 1:** Potential target genes selected for new antifungal drug development

Gene	Biological process	Cytolocalization	PDB template	Organism	*E-value*	PDB sequence identidy (%)
*trr1*	Cell redox homeostasis	Cytoplasm	1ITJ	*Saccharomyces cerevisiae*	3e-115	65
			1VDC	*Arabidopsis thaliana*	1e-94	57
*aur1*	Cellular metabolism	Golgi and membrane	*	*	*	*
*mak5*	Ribosome biogenesis	Nucleolus	1HV8	*Methanocaldococcus jannaschii*	7e-42	30
*chs1*	Cell wall biogenesis/degradation	Membrane	*	*	*	*
*tom40*	Protein transport	Mitochondrion membrane	2QK9	*Homo sapiens*	0.8	34
*kre6*	Cell wall biogenesis/degradation	Golgi apparatus Membrane	2VY0	*Pyrococcus furiosus*	6e-4	32
*fks1*	Cell wall organization/biogenesis	Membrane	1R1M	*Neisseria meningitidis*	0.3	32
*kre2*	Protein mannosilation	Golgi membrane	1S4N	*Saccharomyces cerevisiae*	6e-96	50
*erg6*	Ergosterol biosynthesis	Endoplasmatic reticulum membrane	3BUS	*Lechevalieria aerocolonigenes*	5e-18	32
*rim8*	pH-response regulator	Cytoplasm	3G3L	*Bacteroides fragilis*	3,9	38

* Structure absent in PDB (http://www.rcsb.org/pdb/home/home.do)

Six criteria were used to select the potential targets: 1) be essential or relevant for fungi survival; 2) be present in all analyzed pathogens, therefore allowing a broad spectrum of drug action; 3) be absent in the human genome, therefore avoiding unwanted side effects; 4) be preferentially an enzyme and have the potential for assayability; 5) not be auxotrophic, thereby avoiding host provision of the necessary substrate for the blocked pathway; and 6) have a cellular localization potentially accessible to the drug activity. Applying these criteria, four potential drug targets were identified: *trr1*, *rim8*, *kre2 *and *erg6 *genes. Only *trr1 *and *rim8 *are essential genes, but *kre2 *and *erg6 *are involved in cell viability and survival within the host. In addition, those genes were also identified as potential drug targets in *P. lutzii *isolate Pb01 transcriptome, as described by Felipe and colleagues [21].

The *trr1 *is an essential gene that encodes for the cytoplasmatic enzyme thioredoxin reductase [22]. This protein plays a critical role in maintaining the cell redox status [22] and is part of the complex so-called thioredoxin system, which contains thioredoxin (Trx), thioredoxin reductase (Trr) and NADPH, protecting cells against oxidative stress [23]. Thioredoxin reductase is necessary for the viability of *C. neoformans *[24] and is essential for erythrocytic stages in *Plasmodium falciparum *[25]; it also appears to be essential for growth in *S. aureus *[26]. *S. cerevisiae *strain deleted for *trr1 *gene is hypersensitive to hydrogen peroxide and high temperatures [27,28].

The *rim8 *is also an essential gene that encodes for a protein involved in the proteolytic activation of a transcriptional factor in response to alkaline pH and is located near the plasma membrane [29]. RIM8 (for yeasts) or PalF (for filamentous fungi) protein binds strongly to the C-terminal cytoplasmic tail of the seven transmembrane domains, the putative pH sensor PalH. Alignment of protein sequences suggests structural similarity of RIM8 to mammalian arrestins, but the sequence similarity was restricted to short stretches of amino acid sequences, mostly corresponding to β-strands in arrestin crystal structures [30]. The RIM8 protein performs an essential step in the signaling pathway activating RIM101, which, in turn, regulates alkaline pH-response. This pathway is also involved in the activation of the yeast-to-hyphal transition required for host-pathogen interaction [31].

The *kre2 *gene encodes for the enzyme α-1,2-mannosyltransferase that is located in the Golgi complex. It has a short amino-terminal cytoplasmic domain, a hydrophobic membrane-spanning domain and a large carboxy-terminal catalytic domain [32]. This enzyme is responsible for the addition of the α-1,2-linked mannose residues to O-linked carbohydrates and is also involved in N-linked glycosylation [33-36]. Cell wall-associated proteins are commonly glycosylated and defects in this process may result in protein misfolding, instability, and/or reduced enzymatic activity [36]. Absence of MNT1p in *S. cerevisiae *resulted in the synthesis of truncated O-linked oligosaccharides and this interfered with the functioning and/or synthesis of cell wall compounds [33,34]. Mutants of *C. albicans *that lack CaMNT1 and CaMNT2 have truncated O-mannan, marked reduction in adherence and attenuated virulence [34]. Although CaMNT1p is not essential for viability, MNT1p-mediated O-glycosylation of proteins of *C. albicans *is essential for normal host-fungus interactions [37].

The *erg6 *gene encodes for the enzyme Δ-(24)-Sterol C-methyltransferase that is located in the endoplasmic reticulum. It shows a transmembrane portion and an active site positioned toward the cytoplasm [38,39]. This enzyme catalyzes the attachment of a methyl group acting in a bifurcation point of the ergosterol/cholesterol biosynthesis pathway [40]. In *S. cerevisiae*, *erg6 *mutants showed alteration in membrane fluidity and permeability [41,42]. In *C. albicans*, mutants that do not synthesize Δ-(24)-Sterol C-methyltransferase showed an increase in the plasma membrane permeability, resulting in cells with severely compromised phenotypes [20]. *erg6 *mutants, in *Candida lusitaniae*, showed a severe growth defect and decreased ergosterol content [43].

### Conserved domains in protein sequences and phylogenetic analysis

A multiple protein sequence alignment showed the presence of conserved domains mainly in the catalytic site of the four selected candidates (Additional files 2, 3, 4, 5). The catalytic site of the protein TRR1 contains the four-amino acid-residue sequence Cys-Ala-Thr-Cys [44,22,45], and these two highly conserved cysteine residues (Cys142 and Cys145 in *C. albicans*) are essential for its redox activity (Additional file 2). RIM8 protein alignments showed that in the C-terminal domain, the residue Ile-331 of *A. nidulans*, involved in PalF-PalH receptor binding, was conserved in all the fungi sequences (it is located in position Ile-320 in *P. brasiliensis*). The amino acid residue Ser-86 of *A. nidulans*, present in the N-terminal domain and responsible for PalF-PalH interaction and pH signaling, was replaced by the conserved Cys-75 in *P. brasiliensis*, *B. dermatitidis*, *H. capsulatum*, Cys-76 in *C. immitis *and Cys-77 in *A. fumigatus *(Additional file 3).

The catalytic site of KRE2 proteins contains the conserved amino acid residues His312, His377, Asp350 and Glu318 in *C. albicans*. If those residues are individually replaced, in *C. albicans*, the enzyme activities are fully abolished [46,47]. Our analysis showed highly conserved catalytic domains of all the sequences of the analyzed proteins for all the pathogenic fungi. The domain YNLCHFWSNFEI, previously described as important to the catalysis mechanism, was also conserved in all fungi analyzed (Additional file 4).

The ERG6 protein showed four conserved regions in several sterol methyltransferase (SMT) proteins, including Regions II, III and IV, generally present in AdoMet-dependent enzymes and, Region I, observed in all SMT enzymes [48]. Region I, a highly conserved region rich in aromatic amino acids, contains a signature motif YEXGWG [49]. The mutation of the amino acid residues situated in Region I altered the catalytic behavior of the fungal SMT [50-52]. In addition, specific-site mutation in Region II and Region III of ERG6 protein showed that certain residues (Cys128, Gly129, Pro133 and Ala193 in *S. cerevisiae*) were important to C-methylation activity [52]. All important amino acid residues for ERG6 protein activity were conserved in all fungi genomes analyzed (Additional file 5).

**Figure 1:**
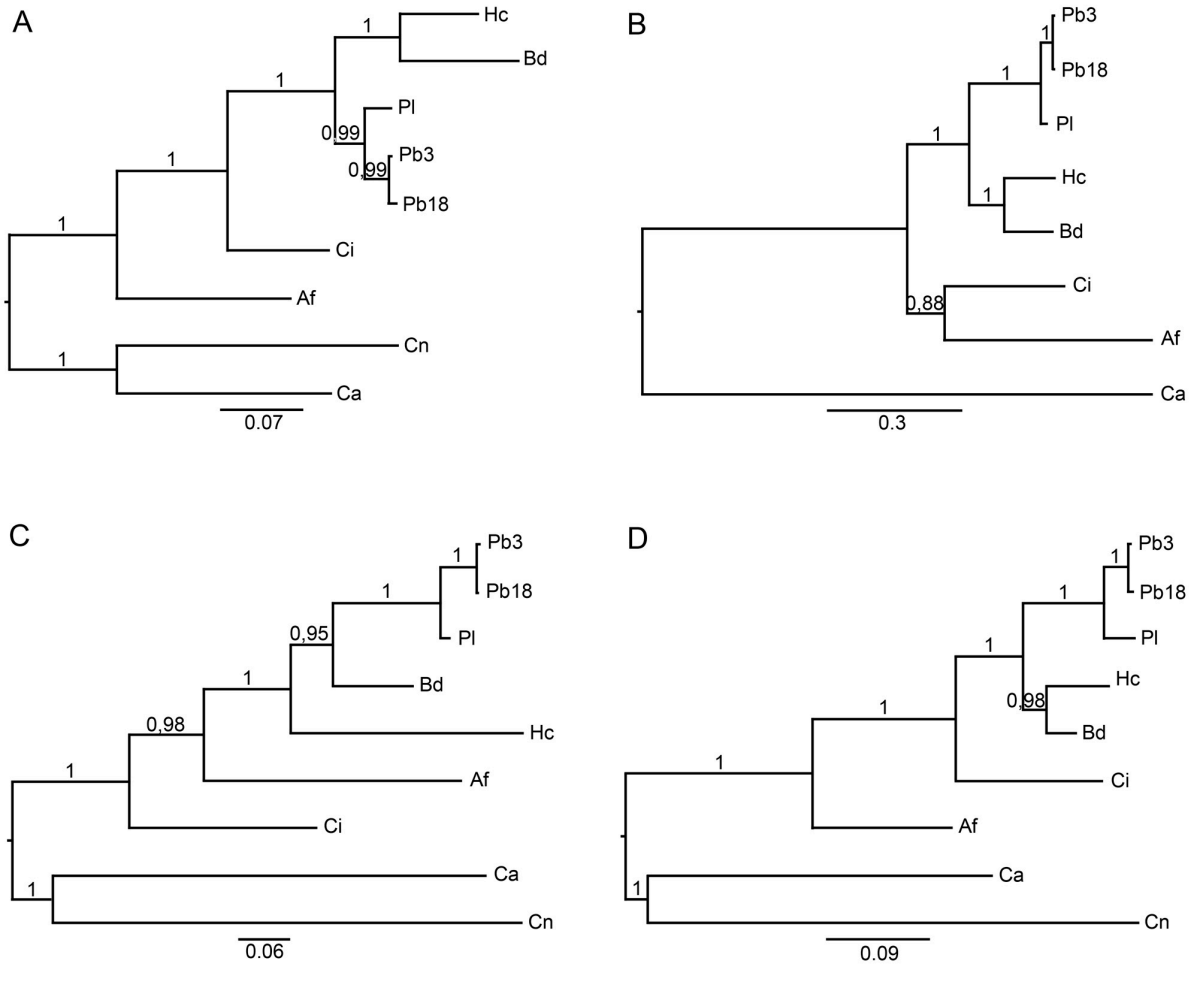
**Phylogenetic analysis between human pathogenic fungi performed by Bayesian analysis**. Phylogenetic trees generated from Bayesian analysis of the genes *trr1 ***(a)**, *rim8 ***(b)**, *kre2 ***(c) **and *erg6 ***(d)**. The length of the vertical lines linking one protein is proportional to the estimated distance between their sequences. The posterior probability values were added to the phylogenetic branches. Af: *Aspergillus fumigatus*, Bd: *Blastomyces dermatitidis*, Ca: *Candida albicans*, Ci: *Coccidioides immitis*, Cn: *Cryptococcus neoformans*, Hc: *Histoplasma capsulatum*, Pl: *Paracoccidioides lutzii*, Pb3: *P. brasiliensis *isolate 3, Pb18: *P. brasiliensis *isolate 18.

The alignments of protein sequences were also used in phylogenetic studies performed by Bayesian analysis to construct phylogenetic trees relating TRR1, RIM8, KRE2 and ERG6 orthologs. The phylogenetic trees showed the evolutionary relationships between the different species used in this work and separated them in different groups (Figure 1). *P. brasiliensis*, *B. dermatitidis*, *H. capsulatum*, *A. fumigatus *and *C. immitis *were clustered apart from *C. albicans *and *C. neoformans*. The posterior probability values were added to the phylogenetic branches and received values near 1, showing the consistency and reliability of these branches. In the four phylogenetic trees, the *P. brasiliensis *isolate Pb01 was separated from the other isolates (Pb3 and Pb18). These findings are in agreement with Teixeira *et al*. 2009 [53], in which 13 single-locus topologies showed that the genus *Paracoccidioides *contains two highly divergent groups. As proposed by Teixeira and colleagues [53], these results reinforce the existence of two species for *Paracoccidioides *genus: *P. brasiliensis *(Pb18 and Pb3) and *P. lutzii *(Pb01).

### Homology modeling of TRR1 and KRE2

In the absence of experimentally solved structures, computational methods were used to predict 3D protein models and provide information regarding protein functions and structures [54]. Homology modeling is efficient in new drug design, from the biological target conception through new drug discovery [55]. Of the four selected potential targets obtained from our comparative genomic analyses, only TRR1 and KRE2 showed a reasonable sequence identity to the templates found in PDB (Table 1). Consequently, we performed the homology modeling only for these two proteins.

According to the BLAST search performed on the entire PDB database, the thioredoxin reductase (TRR1) of *P. brasiliensis *showed good sequence identity with two templates, specifically 3ITJ (PDB ID) of *S. cerevisiae *(65% sequence identity) and 1VDC (PDB ID) of *Arabidopsis thaliana *(57% sequence identity). In the case of α-1,2-mannosyltransferase (KRE2) of *P. brasiliensis*, only the PDB template 1S4N (PDB ID) of *S. cerevisiae *showed a reasonable sequence identity (50%). Starting from the BLAST alignment between *P. brasiliensis *TRR1 and KRE2 proteins with the PDB templates as found above, we manually modified them in order to preserve the secondary structures and the correspondence between cysteine residues (Additional file 6a and 6b).

In these alignments between the target sequences and template structures, a fragment of the C-terminus region of TRR1 (Glu325-Leu358) and of the N-terminus region of KRE2 (Met1-Phe70) did not align. Therefore, a BLAST search with the fragment sequences was performed to verify if these regions had similarity with proteins deposited in the PDB. No confirmation was found, so these fragment terminus regions of TRR1 and KRE2 were removed from the models. This was legitimate, since these fragments are not involved in the active site of the proteins and should not interfere with the virtual screening that we intend to perform using these models.

**Figure 2:**
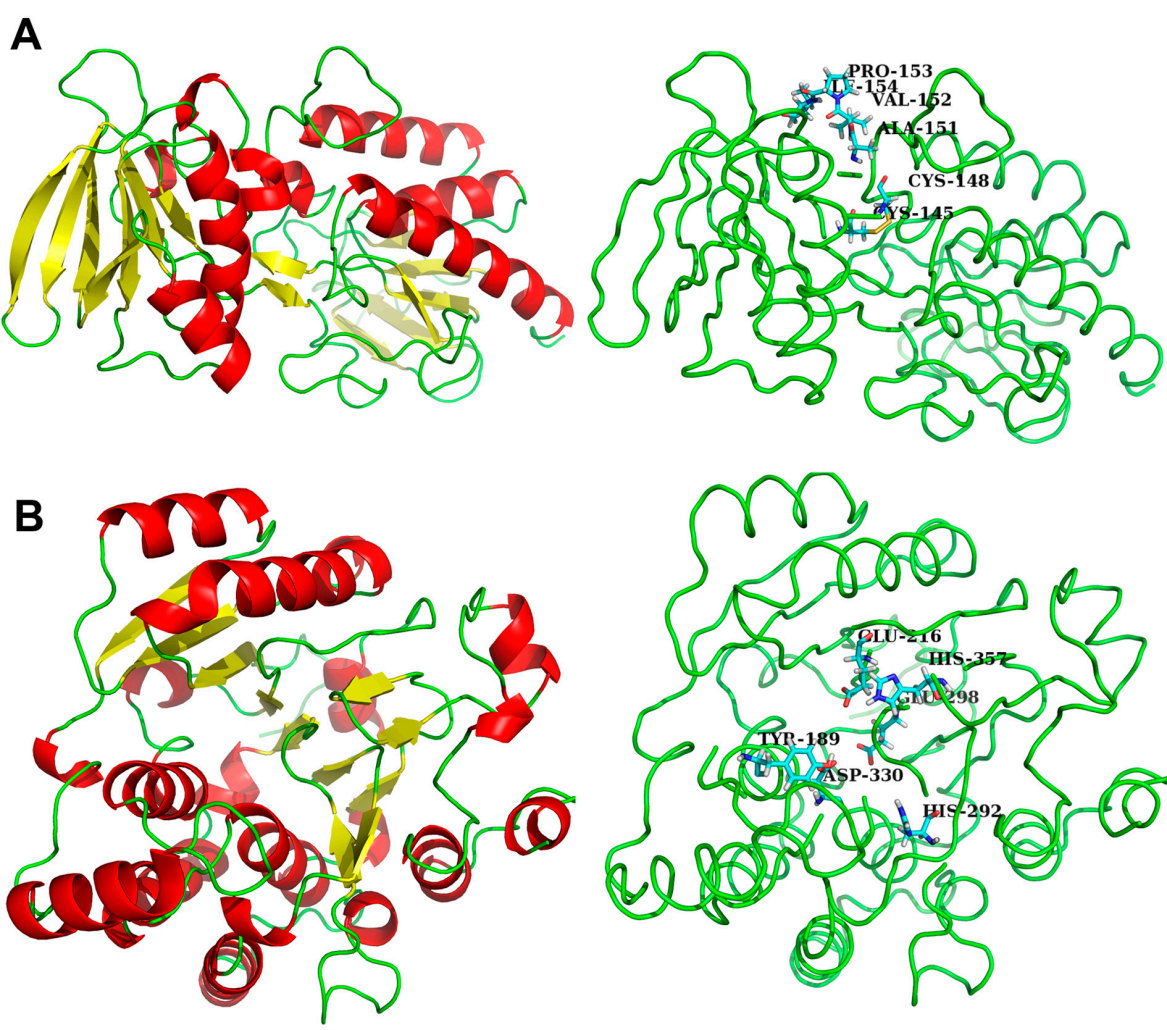
**The predicted tridimensional structure of TRR1 and KRE2 proteins obtained by homology modeling**. The structures of TRR1 **(a) **and KRE2 **(b) **proteins. The α-helix is represented by the color red, the β-sheet is represented by yellow and loops are represented by green. **(a - left panel) **The KRE2 active site presents the conserved residues His292, His357, Asp330 and Glu298. (Mutation of these residues abolished the protein activity in *C. albicans.*) Additional residues found in the KRE2 active site include Glu216, which interacts with the metal ion Mn^2+ ^and creates the reactive nucleophylic center for the glycosyltransferase reaction, and Tyr189, which coordinates the donor and acceptor binding that allows the transfer of the mannose to the growing oligosaccharide. **(b - right panel) **The TRR1 protein is composed of two domains that comprise the binding sites of NADPH and FAD. The NADPH binding domain contains the active Cys145 and Cys148 residues. Other important residues of the TRR1 active site are Ala151, Val152, Pro153 and Ile154 that form a hydrophobic region in the NADPH binding domain.

The refinement of the homology models was obtained through molecular mechanics optimization; the stable structures of TRR1 and KRE2 are displayed in Figure 2. Figure 2a shows that the enzyme TRR1 has 9 helices and 17 sheets. Figure 2b shows that the enzyme KRE2 has 18 helices and 11 sheets.

The TRR1 monomeric protein of *S. cerevisiae *is composed of two domains that form the binding sites of NADPH and FAD similar to plants. The FAD molecule is bound to the *S. cerevisiae *TRR1 protein and is stabilized by interactions with the residues Pro13, Glu33, Gln45, Asn54, Gln136, Asp288 and Gln296. The NADPH binding domain contains active cysteine residues and is linked to the FAD domain by a short β-sheet [56,57]. Figure 2a shows the two domains in the *P. brasiliensis *TRR1 model.

In KRE2 protein, the catalytic mechanism of the active site involves nucleophylic substitutions mediated by acidic amino acid residues and an essential Mn^2+ ^cofactor. Heterologous expression of site-specific mutants of *C. albicans *MNT1 protein in *Pichia pastoris *confirmed the nature of a nucleophilic reaction center, where the two conserved histidines (His292 and His357 in *P. brasiliensis*) that coordinated the metal ion cofactor Mn^2+ ^and created the reactive nucleophylic center required the nonprocessing, GDP-mannose-dependent, retaining glycosyltransferase reaction [46]. However, Lobsanov and colleagues [47] examined the structure and catalysis mechanism of *S. cerevisiae *KRE2 enzyme (1S4N template) by crystallography and proposed a novel mechanism for this interaction, and the precise function for the conserved amino acids was determined by site-direct mutagenesis by Thomson and colleagues [46]. The proposed mechanism of retaining glycosyltransferases such as CaMNT1p involves a two-step displacement. The first step involves attack on the sugar anomeric center by one of the carboxylates, and then a second carboxylate acts as the active site nucleophilic to displace the GDP from the sugar nucleotide, leading to formation of a glycosyl-enzyme intermediate. The metal ion Mn^2+ ^is coordinated by a direct interaction of the residue glutamate (Glu216 in KRE2 protein of *P. brasiliensis*) as shown in Figure 2b, right panel. Transfer of the mannose to the growing oligosaccharide is completed by displacement of the enzyme from the intermediate by the hydroxyl group of the acceptor [46]; in *C. albicans *KRE2 protein, tyrosine (Tyr209) coordinates the donor and acceptor binding from the N-terminal domain and plays a role in the catalysis [47].

## Conclusions

We reported a comparative genomic strategy to provide a list of potential antifungal drug targets for the human pathogenic fungi *P. brasiliensis*, *P. lutzii*, *A. fumigatus*, *B. dermatitidis*, *C. albicans*, *C. immitis*, *C. neoformans *and *H. capsulatum*. The preferred profile for fungal targets was proteins conserved among these fungi, but absent in the human genome, aiming to minimize the potential toxic side effects exerted by pharmacological inhibition of the cellular targets. In general, the potential drug targets were selected following the criteria of essentiality, presence in all human pathogenic fungi considered here, absence in humans, be preferentially an enzyme, not be auxotrophic and have accessible cell localization.

*In silico *and manual mining provided four genes as potential drug targets: *trr1 *that encodes for thioredoxin reductase, *rim8 *that encodes for a protein involved in the proteolytic activation of a transcriptional factor in response to alkaline pH, *kre2 *that encodes for α-1,2-mannosyltransferase and *erg6 *that encodes for Δ-(24)-Sterol C-methyltransferase. The increase in structural databases allows the satisfactory prediction of structures by theoretical methods, with advantages over more costly experimental methods. We performed the homology modeling for the potential targets that were identified to have a known 3D structure and that showed good sequence identity to the templates found in PDB, TRR1 and KRE2. In the absence of structures solved experimentally, the available homology modeling tools were extremely useful for the structural prediction of the TRR1 and KRE2 proteins. From this first step of post-genomic analysis, we obtained relevant information for future technological development. Moreover, these results are being used to virtually screen chemical libraries, which are under progress, generating new perspectives on technological development and innovation of antifungal agents to these human pathogens.

## Methods

### Comparative analysis of human pathogenic fungi genomes and drug target selection

The identification of potential drug targets was based on 55 genes experimentally confirmed as essential for *Candida albicans *[17] or *Aspergillus fumigatus *[18]. In these cases, the genes were experimentally confirmed as essential and represent a large spectrum of biological functions, such as cellular metabolism, cell wall organization and biogenesis, ergosterol biosynthesis, ribosomal biogenesis and post-translational modification of protein [17,18]. Other 2 genes (*kre2 *and *erg6*) were added to the initial screening since they were described as potential drug targets [21].

The 57 gene sequences of were retrieved from the GenBank databases (http://www.ncbi.nlm.nih.gov/) and were used to screen the *P. brasiliensis *Pb01 transcriptome database (https://helix.biomol.unb.br/Pb/) using blastn. The sequences of *Paracoccidioides lutzii *isolate Pb01 were not applied as a filter since all 57 genes were present and expressed in its genome/transcriptome. Subsequently, the presence of these genes in the 2 isolates of *P. brasiliensis *(Pb3 and Pb18) was confirmed. Using the isolate Pb01 sequence, released by Broad Institute (http://www.broad.mit.edu/), the orthologs search in other pathogenic fungi (*A. fumigatus *Af293, *B. dermatitidis *ER3, *C. albicans *WO1, *C. immitis *H538.4, *C. neoformans *serotype B, *H. capsulatum *NAm1) and human genome was completed using blastx because we have focused in the development of a new antifungal not only for the *Paracoccidioides species *but for all medically important fungi. The cut-off established for determining the presence of an ortholog was a maximum E-value of 0.00001 (1e-5). A manual curation was performed to select the potential drug targets following the criteria of essentiality, be present in pathogenic fungi, be absent in humans, be preferentially an enzyme, not be auxotrophic and have accessible cell localization.

### Multiple alignments of the orthologs genes and phylogenetic analysis

Sequences were aligned by the ClustalW using dynamic programming and hierarchical methods [58] available in the BioEdit software [59]. The program identified conserved regions in the protein sequences between orthologs target genes by multiple sequence alignments.

The sequences were also used for phylogenetic analysis by Bayesian inference using Mr. Bayes software, version 3.1.2 [60]. Detected gaps in sequence alignments were considered missing data and coded in terms of presence or absence. The amino acid substitution model selected was JTT [61]. The Markov Chain Monte Carlo (MCMC) was initiated from a random tree and processed for 1000000 generations; sample trees were retrieved every 1000 generations. Log-likelihood values were plotted against the generation number, and the first 25% of samples were discarded ("*burn-in*"). The remaining samples were used to determine the distribution of posterior probability values. Phylogenetic trees were produced with the help of the Treeview and Figtree 1.0 software.

### Protein structure prediction

There is no crystallographic structure presently available for TRR1 and KRE2 of *P. brasiliensis *and also for the other pathogenic fungi. Therefore, the 3D structures of TRR1 and KRE2 of *P. brasiliensis *were constructed by homology modeling based on known structures with high percentage of identity in amino acid sequences. We have initially modeled *P. brasiliensis *proteins but it will be similar for the other pathogenic fungi since the sequences of the proteins are highly conserved. The known template structures were searched in the PDB. There were two templates for TRR1 protein: 3ITJ (PDB ID) of *S. cerevisiae *and 1VDC (PDB ID) of *A. thaliana*. There was one template for KRE2 protein: 1S4N (PDB ID) of *S. cerevisiae*. The templates structures for ERG6 and RIM8 showed low sequence identity, then not allowed the construction of 3D structures for these proteins by molecular modeling. The amino acid residue sequences of TRR1 and KRE2 were compared with the primary sequences of the structures deposited in the PDB using the BLAST program. The homologous sequences allowed the construction of a 3D model of TRR1 and KRE2 using the homology module of the Insight II software package (Biosym/MSI, San Diego, Accelrys Inc. 2001). Briefly, the target sequences were aligned with the template structures, and coordinates from the templates were transferred to the targets TRR1 and KRE2. For model optimization, the backbone atoms of the structures were initially frozen and only the side chains were allowed to move for a selective minimization by conjugate gradient method. A second selective minimization, also by conjugate gradient method, was performed with only atoms of the complementary determining region (CDR) loops moving. The last minimization was performed by Steepest-descent method with all atoms of the structure relaxed, resulting in whole, refined 3D structures. The molecular visualization was performed by PyMOL open-source software version 0.99rc6 (Delano Scientific LLC, 2006).

## Authors' contributions

AA, NM, MF and EK planned and designed the study, developed the experiments and completed the data analysis, wrote the main draft of the paper and supported the preparation of the figures and tables. BM participated in the homology modeling experiments and helped in the manuscript editing. MT participated in the phylogenetic analysis. All authors read and approved the final manuscript

## Supplementary Material

Additional file 1 **Essential genes found in *C. albicans *and/or *A. fumigatus *and orthologs in other human pathogenic fungi**. Click here for file

Additional file 2 **Amino acid alignment between conserved protein residues of TRR1, in the human pathogenic fungi**. Amino acid sequence analysis of TRR1 protein. Af: *Aspergillus fumigatus*, Bd: *Blastomyces dermatitidis*, Ca: *Candida albicans*, Ci: *Coccidioides immitis*, Cn: *Cryptococcus neoformans*, Hc: *Histoplasma capsulatum*, Pb01: *Paracoccidioides brasiliensis *isolate 01, Pb3: *P. brasiliensis *isolate 3, Pb18: *P. brasiliensis *isolate 18. Positions of identity are indicated with *asterisks*, a *semicolon *indicates conserved substitutions, and a *dot *shows a semi-conservative substitution. Click here for file

Additional file 3 **Amino acid alignment between conserved protein residues of RIM8, in the human pathogenic fungi**. Amino acid sequence analysis of RIM8 protein. Af: *Aspergillus fumigatus*, Bd: *Blastomyces dermatitidis*, Ca: *Candida albicans*, Ci: *Coccidioides immitis*, Cn: *Cryptococcus neoformans*, Hc: *Histoplasma capsulatum*, Pb01: *Paracoccidioides brasiliensis *isolate 01, Pb3: *P. brasiliensis *isolate 3, Pb18: *P. brasiliensis *isolate 18. Positions of identity are indicated with *asterisks*, a *semicolon *indicates conserved substitutions, and a *dot *shows a semi-conservative substitution. Click here for file

Additional file 4 **Amino acid alignment between conserved protein residues of KRE2, in the human pathogenic fungi**. Amino acid sequence analysis of KRE2 protein. Af: *Aspergillus fumigatus*, Bd: *Blastomyces dermatitidis*, Ca: *Candida albicans*, Ci: *Coccidioides immitis*, Cn: *Cryptococcus neoformans*, Hc: *Histoplasma capsulatum*, Pb01: *Paracoccidioides brasiliensis *isolate 01, Pb3: *P. brasiliensis *isolate 3, Pb18: *P. brasiliensis *isolate 18. Positions of identity are indicated with *asterisks*, a *semicolon *indicates conserved substitutions, and a *dot *shows a semi-conservative substitution. Click here for file

Additional file 5 **Amino acid alignment between conserved protein residues of ERG6, in the human pathogenic fungi**. Amino acid sequence analysis of ERG6 protein. Af: *Aspergillus fumigatus*, Bd: *Blastomyces dermatitidis*, Ca: *Candida albicans*, Ci: *Coccidioides immitis*, Cn: *Cryptococcus neoformans*, Hc: *Histoplasma capsulatum*, Pb01: *Paracoccidioides brasiliensis *isolate 01, Pb3: *P. brasiliensis *isolate 3, Pb18: *P. brasiliensis *isolate 18. Positions of identity are indicated with *asterisks*, a *semicolon *indicates conserved substitutions, and a *dot *shows a semi-conservative substitution. Click here for file

Additional file 6 **Manual alignments performed between *P. brasiliensis *proteins and the PDB templates**. **(a) ***TRR1 protein and templates *3ITJ*and *1VDC. The boxes represent the template regions that were used as references for the homology modeling of TRR1 protein. In the alignment between TRR1 protein and the templates, the big boxes indicate that the 1VDC template was used as reference and the small box indicates that the reference was the 3ITJ template. Considering the global alignment, 3ITJ is the best template to use as a reference to perform the homology modeling of *P. brasiliensis *TRR1 protein. However, some regions of the 1VDC template present identical amino acids to *P. brasiliensis *TRR1 protein, and those are different in the 3ITJ template. The following colors represent amino acids: white (identical amino acids between TRR1 protein and the two templates), green (identical amino acids between TRR1 protein and 1VDC template), red (identical amino acids between TRR1 protein and 3ITJ template), orange (unique amino acids in 3ITJ template), dark blue (unique amino acids in 1VDC template), and light blue (unique amino acids in TRR1 protein). The cysteine residues that form the disulfide bonds are conserved between TRR1 protein and the two templates. **(b) ***KRE2 protein and template*IS4N. The boxes represent the template regions that were used as references for the homology modeling of KRE2 protein. In the alignment between KRE2 protein and the 1S4N template, the boxes indicate the regions that were used as references for 3D structure construction of KRE2. The following colors represent amino acids: white (identical amino acids between KRE2 protein and the template), purple (similar amino acids between KRE2 protein and the template), red (unique amino acids in KRE2 protein), and light blue (unique amino acids in 1S4N template). The cysteine residues that form the disulfide bonds are conserved between KRE2 protein and the template. Click here for file
