# Supplementary material for: Comparative genomics allowed the identification of drug targets against human fungal pathogens
Source: BMC Genomics. 2011 Jan 27;12:75. doi: 10.1186/1471-2164-12-75 (PMC3042012; doi:10.1186/1471-2164-12-75)
Supplement: Additional file 2 — Amino acid alignment between conserved protein residues of TRR1, in the human pathogenic fungi. Amino acid sequence analysis of TRR1 protein. Af: Aspergillus fumigatus, Bd: Blastomyces dermatitidis, Ca: Candida albicans, Ci: Coccidioides immitis, Cn: Cryptococcus neoformans, Hc: Histoplasma capsulatum, Pb01: Paracoccidioides brasiliensis isolate 01, Pb3: P. brasiliensis isolate 3, Pb18: P. brasiliensis isolate 18. Positions of identity are indicated with asterisks, a semicolon indicates conserved substitutions, and a dot shows a semi-conservative substitution. [file 1471-2164-12-75-S2.PDF]

FAD binding domain

```
Trr1Pb3     SETVSRVDLSCRPFKLWKEFSDGPDDAPAHTADALIIATGANARRLDLPGEQQYWQNGIS 108
Trr1Pb18    SETVSRVDLSCRPFKLWKEFSDGPDDAPAHTADALIIATGANARRLDLPGEQQYWQNGIS 147
Trr1Pb01    SETVSRVDLSCRPFKLWKEFSDGPDDAPAHTADALIVATGANARRLDLPGEQQYWQNGIS 143
Trr1Hc      TETVSRIDLSSRPFKLWKEYADGPNDGPAHTTDAIIIATGANARRLDLPGEERYWQNGIS 108
Trr1Bd      TETVSRVDLSSRPFKVWKEYSDGPNDAAAHTADALIIATGANARRLDLPGEQQYWQNGIS 180
Trr1Ci      TETISRVDLSSRPFKLWKEWSDGPDDAPAHTADALIIATGANARRLDLPGEDKYWQNGIS 143
Trr1Af      TETISRVDLSSKPFKLWTEWNDGPDKEPACTADAVIIATGANARRLNLPGEETYWQNGIS 162
Trr1Ca      TETISKVDFSKRPFKLWTEWN---EDAEPITTDAVIIATGASAKRMHLPGEDTYWQQGIS 140
Trr1Cn      TETVARVDLSVRPFKYWTEGEE--EEHEFMTADTIIMATGASAKRLFLPGEDTYWQSGIS 167
            :**::::*:* :*** *.*      :.    *:*::*:****.*:*: ****: ***.***

Trr1Pb3     ACAVCDGAVPIFRNKPLFVIGGGDSAAEEAMFLTKYGSKVTVLVRRDKLRASKTMAKRLL 168
Trr1Pb18    ACAVCDGAVPIFRNKPLFVIGGGDSAAEEAMFLTKYGSKVTVLVRRDKLRASKTMAKRLL 207
Trr1Pb01    ACAVCDGAVPIFRNKPLFVIGGGDSAAEEAMFLTKYGSKVTVLVRRDKLRASKTMAKRLL 203
Trr1Hc      ACAVCDGAVPIFRNKPLFVIGGGDSAAEEAMFLTKYGSKVTVLVRKDKLRASKTMAKRLL 168
Trr1Bd      ACAVCDGAVPIFRNKPLFVIGGGDSAAEEAMFLTKYGSKVTVLVRKDKLRASKTMAKRLL 240
Trr1Ci      ACAVCDGAVPIFRNKPLFVIGGGDSAAEEAMFLTKYGSSVTVLVRRDKLRASKTMATRLL 203
Trr1Af      ACAVCDGAVPIFRNKPLYVIGGGDSAAEEAMFLAKYGSSVTVLVRRDKLRASKAMAKRLL 222
Trr1Ca      ACAVCDGAVPIFRNNPLAVIGGGDSACEEAIFLTKYASKVFLLVRRDVLRASTIMQKRVT 200
Trr1Cn      ACAVCDGAVPIFRQKPLAVIGGGDSAAEEATYLTKYGSHVYVLVRRDELRASKIMAKRLT 227
            *************::** ********.*** :*:**.* * :***:* ****. * .*:

Trr1Pb3     ANPKVEVKFNTVAVEVQGEPAPRGLMTHLKIKNVVSGGEEVVPANGLFYAVGHDPATALV 228
Trr1Pb18    ANPKVEVKFNTVAVEVQGEPAPRGLMTHLKIKNVVSGGEEVVPANGLFYAVGHDPATALV 267
Trr1Pb01    VNPKVEVKFNTVAVEVQGEPAPRGLVTHLKIKNVVSGVEEVVPANGLFYAVGHDPATALV 263
Trr1Hc      ANPKVEVKFNTVALEVLGDPAPRGLMTHLKIKDVVSGAEEVVPANGLFYAVGHDPATALV 228
Trr1Bd      ANPKVEVKFNTVALEVLGEPEPRGLMTHLKIKNVVSGNEEVVPANGLFYAVGHDPATALV 300
Trr1Ci      NHPKVEVKFNTVAVEVQGEPQPRGLMTHLKIKNVVTGNEEVVPANGLFYAVGHEPATGLI 263
Trr1Af      AHPKVTVRFNTVATEVLGEKKPNGLMTHLRIKNTVTGEEEIVDANGLFYAVGHDPATALV 282
Trr1Ca      NNEKIEVLWNTEALEAKGDGK---LLKSLRIVNNKTKKEKDLQVNGLFYAIGHIPATKIF 257
Trr1Cn      SHPKVTVLWNTVATEAKGDGE---VLTSLTIKDTKTGKIRDLPVNGLFYAIGHEPATALV 284
             : *: * :** * *. *:     ::. * * :  :    . : .******:** *** :.
```
