# Supplementary material for: Comparative genomics allowed the identification of drug targets against human fungal pathogens
Source: BMC Genomics. 2011 Jan 27;12:75. doi: 10.1186/1471-2164-12-75 (PMC3042012; doi:10.1186/1471-2164-12-75)
Supplement: Additional file 3 — Amino acid alignment between conserved protein residues of RIM8, in the human pathogenic fungi. Amino acid sequence analysis of RIM8 protein. Af: Aspergillus fumigatus, Bd: Blastomyces dermatitidis, Ca: Candida albicans, Ci: Coccidioides immitis, Cn: Cryptococcus neoformans, Hc: Histoplasma capsulatum, Pb01: Paracoccidioides brasiliensis isolate 01, Pb3: P. brasiliensis isolate 3, Pb18: P. brasiliensis isolate 18. Positions of identity are indicated with asterisks, a semicolon indicates conserved substitutions, and a dot shows a semi-conservative substitution. [file 1471-2164-12-75-S3.PDF]

```
Rim8Pb3         VILKVVKPIRITHLVLCLHGYVKVFKNTVAPGEGSEESGFLGPGRGRRKSEYLGNGFATL 118
Rim8Pb18        VILKVVKPIRITHLVLCLHGYVKVFKNTVAPGEGSEESGFLGPGRGRRKSEYLGNGFATL 118
Rim8Pb01        VILKVVKPIRITHLVLCLHGYVKVFKNTVAPGEGSEESGFLGPGRGRRNSEYLGNGFATL 118
Rim8Hc          VVLKVVKPIRITHLVLCLHGYVRVYKTTVAPGDVSDASGFLGPGRGKRNGEYLGNGFATL 118
Rim8Bd          VVLKVVKPVRITHLVLCLHGYVKVYKTAVAPGDALEESGFLGPGRGKRYGEYLGNGFASL 118
Rim8Ci          VCLVVVKPVRITHLVVCLHGYAKVYKNPVAPGETAEDSGFPGTGRGRRNGEYLGNGLATL 119
Rim8Af          VALTVVRPVRITHLVVCLHGYVKVFKNTVPSGETDPDLGFLGPGRGRRGPEYLGNGLATL 120
Rim8Ca          VVLISKKNLANIVITLSLVGFIKINASSHSKLRPLKHTLFDYTIKIYGKDEEE------- 108
                * *   : :    :.:.* *: ::  .. .         *  . :     *

Rim8Pb3         KTIKAKTELLRPGGVPGDILPVKITIQHTKPIRSPNGIIITLYRQGRIDYHPSIPVGPAE 354
Rim8Pb18        KTIKAKTELLRPGGVPGDILPVKITIQHTKPIRSPNGIIITLYRQGRIDYHPSIPVGPAE 354
Rim8Pb01        KTIKATTELLRPGGVPGDILPVKITIQHTKPIRSPNGIIITLYRQGRIDYHPSIPVGPAE 354
Rim8Hc          RTITATTELSRPGGVPGDVLPVKITIQHTKPIRSPNGIIITLYRQGRIDYHPAIPVGYSE 354
Rim8Bd          RTIKATTELSRPGGVPGDVLPVKITIQHTKPIRSPNGIIITLYRQGRIDLHPAIPVGHTD 354
Rim8Ci          QEITATTKVLRAGVLPGDLLPVNISIKHTKPIRSPNGVIITLYRQGRIDMYPQLPIGTPE 352
Rim8Af          KTITAKTELLRAGVLPGETLPIVITINHCKQVRSAHGIIVTLYRQGRIDLHPAIPIGTTA 356
Rim8Ca          PTIKAILEVPQRGYLRGESIPIKLSINHLRKIQDFNGIIITFVRVCRLDNGPDGVVES-- 324
                  *.*  :: : * : *: :*: ::*:* : ::. :*:*:*: *  *:*  *   :
```
