# Supplementary material for: Comparative genomics allowed the identification of drug targets against human fungal pathogens
Source: BMC Genomics. 2011 Jan 27;12:75. doi: 10.1186/1471-2164-12-75 (PMC3042012; doi:10.1186/1471-2164-12-75)
Supplement: Additional file 5 — Amino acid alignment between conserved protein residues of ERG6, in the human pathogenic fungi. Amino acid sequence analysis of ERG6 protein. Af: Aspergillus fumigatus, Bd: Blastomyces dermatitidis, Ca: Candida albicans, Ci: Coccidioides immitis, Cn: Cryptococcus neoformans, Hc: Histoplasma capsulatum, Pb01: Paracoccidioides brasiliensis isolate 01, Pb3: P. brasiliensis isolate 3, Pb18: P. brasiliensis isolate 18. Positions of identity are indicated with asterisks, a semicolon indicates conserved substitutions, and a dot shows a semi-conservative substitution. [file 1471-2164-12-75-S5.PDF]

```
                                                      Region I
Erg6Pb3    SADETEEVREARRAEYATLTRHYYNLATDFYEHGWGSSFHFCRFAYGEPFRQSIARHEHY 118
Erg6Pb18   SADETEEVREARRAEYATLTRHYYNLATDFYEHGWGSSFHFCRFAYGEPFRQSIARHEHY 118
Erg6Pb01   SADETEEVREARRAEYATLTRHYYNLATDFYEYGWGSSFHFCRFAYGEPFRQSIARHEHY 118
Erg6Hc     STEETDEIREARRVEYATLTRHYYNLATDFYEYGWGSSFHFCRFAYGEPFRQAIARHEHY 118
Erg6Bd     SAEETEEIRQARRAEYATLTRHYYNLATDFYEYGWGSSFHFCRFAYGEPFRQAIARHEHY 118
Erg6Af     AEDETEETRAARRAEYATLTRHYYNLATDLYEYGWGTSFHFCRFAQGEPFYQAIARHEHY 118
Erg6Ca     SKDDEEK----RLNDYSQLTHHYYNLVTDFYEYGWGSSFHFSRYYKGEAFRQATARHEHF 113
Erg6Cn     SANDNDTHRANRLDQYTEVVNGYYDGATELYEYGWSESFHFCRFYKGEAFLQALARHEHY 81
Erg6Ci     ARDDTEDERDGRISKYMSLVNSYYDLATDIYEEAWAQSFHLCRFAIGEPLQQALARHEHY 120
           : :: :     *   .*  :.. **: .*::** .*. ***:.*:  **.: *: *****:

                      Region II
Erg6Pb3    LAHWVGLKENQLVLDVGCGVGGPAREIVKFAGVNVIGLNNNDYQIDRAVHYATKEGLSDK 178
Erg6Pb18   LAHWVGLKENQLVLDVGCGVGGPAREIVKFAGVNVIGLNNNDYQIDRAVHYATKEGLSNK 178
Erg6Pb01   LAHWIGLKENQLVLDVGCGVGGPAREIVKFTGVNVIGLNNNDYQIDRAIHYATKEGLSDK 178
Erg6Hc     LAHQIGLKEDQLVLDVGCGVGGPAREMVKFAGVNIVGLNNNDYQIDRATHYAAKEGLSHK 178
Erg6Bd     LAHQIGLREDQLVLDVGCGVGGPAREIVKFAGVNVIGLNNNDYQIDRATHYAAKEGLSHK 178
Erg6Af     LAHQMGIKEGMKVLDVGCGVGGPAREIVKFTDANVVGLNNNDYQIERATRYAEREGLSHK 178
Erg6Ca     LAHKMNLNENMKVLDVGCGVGGPGREITRFTDCEIVGLNNNDYQIERANHYAKKYHLDHK 173
Erg6Cn     LASMMQLKPGMRVLDVGCGVGGPAREIARFSDANIVGINNNDFQIGRATAKSKKAGLSDK 141
Erg6Ci     LAYRINLSPDMHVLDVGCGVGRPAREMATFTGCNVVGLNNNGYQIQRAKAHAERERLSHK 180
           **   : :  .  ********* *.**:. *:. :::*:***.:** **   : :  *..*

                          Region III                  Region IV
Erg6Pb3    LRFVKGDFMQMS--FEPETFDAVYAIEATVHAPSLEGIYSEIYRVLKPGGTFGVYEWVMT 236
Erg6Pb18   LRFVKGDFMQMS--FEPETFDAVYAIEATVHAPSLEGIYSEIYRVLKPGGTFGVYEWVMT 236
Erg6Pb01   LRFTKGDFMQMS--FEPETFDAVYAIEATVHAPSLEGIYSEIYRVLKPGGTFGVYEWVMT 236
Erg6Hc     LRFTKGDFMQMS--FEPETFDAAYAIEATVHAPSLEGAYSEIYRVLKPGGTFGVYEWVMT 236
Erg6Bd     LRFTKGDFMQMS--FEPETFDAVYAIEATVHAPSLEGVYSQIYRVLKPGGTFGVYEWVMT 236
Erg6Af     LSFVKGDFMQMK--FPDNSFDAVYAIEATVHAPDLEGVYKEIFRVLKPGGVFGVYEWLMT 236
Erg6Ca     LSYVKGDFMQMD--FEPESFDAVYAIEATVHAPVLEGVYSEIYKVLKPGGVFGVYEWVMT 231
Erg6Cn     VSFVKGDFMKLSEQFGENSFDAIYAIEATCHAPDFEGIYGEIFKCLKPGGVFGVYEWCMT 201
Erg6Ci     VSFVKGDFMHLE--FPENSFDAAYAIEATVHAPSLQGVYEQIYRVLKPGGTFGVYEWVMT 238
           . .  ******..   *  ..*** ****** *** ..* * .*.. ***** ****** **
```
