# Supplementary material for: Comparative genomics allowed the identification of drug targets against human fungal pathogens
Source: BMC Genomics. 2011 Jan 27;12:75. doi: 10.1186/1471-2164-12-75 (PMC3042012; doi:10.1186/1471-2164-12-75)
Supplement: Additional file 6 — Manual alignments performed between P. brasiliensis proteins and the PDB templates. (a) TRR1 protein and templates 3ITJand 1VDC. The boxes represent the template regions that were used as references for the homology modeling of TRR1 protein. In the alignment between TRR1 protein and the templates, the big boxes indicate that the 1VDC template was used as reference and the small box indicates that the reference was the 3ITJ template. Considering the global alignment, 3ITJ is the best template to use as a reference to perform the homology modeling of P. brasiliensis TRR1 protein. However, some regions of the 1VDC template present identical amino acids to P. brasiliensis TRR1 protein, and those are different in the 3ITJ template. The following colors represent amino acids: white (identical amino acids between TRR1 protein and the two templates), green (identical amino acids between TRR1 protein and 1VDC template), red (identical amino acids between TRR1 protein and 3ITJ template), orange (unique amino acids in 3ITJ template), dark blue (unique amino acids in 1VDC template), and light blue (unique amino acids in TRR1 protein). The cysteine residues that form the disulfide bonds are conserved between TRR1 protein and the two templates. (b) KRE2 protein and templateIS4N. The boxes represent the template regions that were used as references for the homology modeling of KRE2 protein. In the alignment between KRE2 protein and the 1S4N template, the boxes indicate the regions that were used as references for 3D structure construction of KRE2. The following colors represent amino acids: white (identical amino acids between KRE2 protein and the template), purple (similar amino acids between KRE2 protein and the template), red (unique amino acids in KRE2 protein), and light blue (unique amino acids in 1S4N template). The cysteine residues that form the disulfide bonds are conserved between KRE2 protein and the template. [file 1471-2164-12-75-S6.PDF]

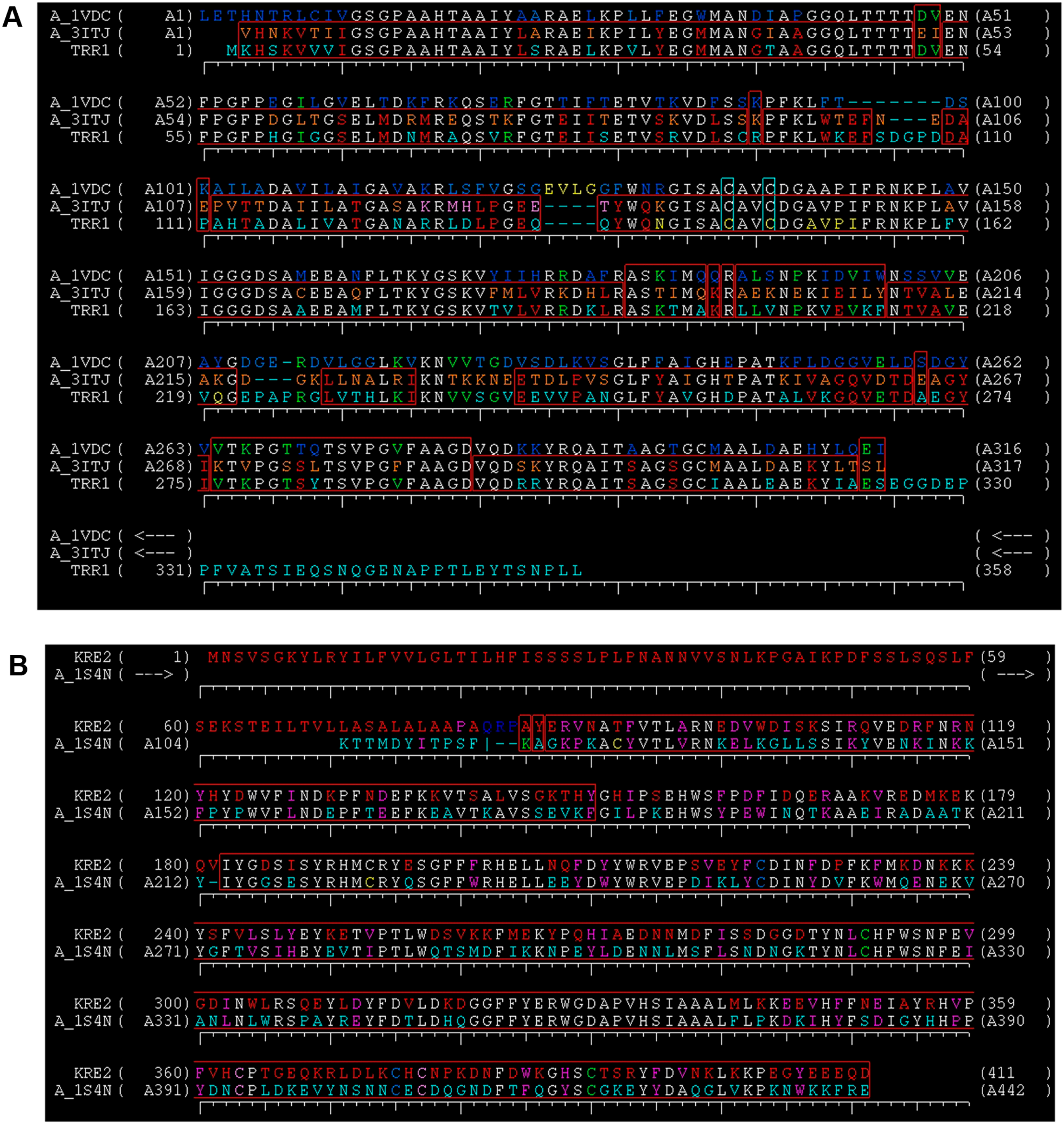
A
A_1VDC (A1) LETHNTRLCIVGSGPAAHTAAIYAARAELKPLLFEGWMANDIAPGGQLTTTTDVEN (A51)
A_3ITJ (A1) VHNKVTIIGSGPAAHTAAIYLARAEIKPILYEGMMANGIAAGGQLTTTTEIEN (A53)
TRR1 (1) MKHSKVVVIGSGPAAHTAAIYLSRAELKPVLYEGMMANGTAAGGQLTTTTDVEN (54)
A_1VDC (A52) FPGFPEGILGVELTDKFRKQSERFGTTIFTETVTKVDFSSKPFKLFT-------DS (A100)
A_3ITJ (A54) FPGFPDGLTGSELMDRMREQSTKFGTEIITETVSKVDLSSKPFKLWTEFN---EDA (A106)
TRR1 (55) FPGFPHGIGGSELMDNMRAQSVRFGTEIISETVSRVDLSCRPFKLWKEFSDGPDDA (110)
A_1VDC (A101) KAILADAVILAIGAVAKRLSFVGSGEVLGGFWNRGISACAVCDGAAPIFRNKPLAV (A150)
A_3ITJ (A107) EPVTTDAIILATGASAKRMHLPGEE----TYWQKGISACAVCDGAVPIFRNKPLAV (A158)
TRR1 (111) PAHTADALIVATGANARRLDLPGEQ----QYWQNGISACAVCDGAVPIFRNKPLFV (162)
A_1VDC (A151) IGGGDSAMEEANFLTKYGSKVYIIHRRDAFRASKIMQQRALSNPKIDVIWNSSVVE (A206)
A_3ITJ (A159) IGGGDSACEEAQFLTKYGSKVFMLVRKDHLRASTIMQKRAEKNEKIEILYNTVALE (A214)
TRR1 (163) IGGGDSAAEEAMFLTKYGSKVTVLVRRDKLRASKTMAKRLLVNPKVEVKFNTVAVE (218)
A_1VDC (A207) AYGDGE-RDVLGGLKVKNVVTGDVSDLKVSGLFFAIGHEPATKFLDGGVELDSDGY (A262)
A_3ITJ (A215) AKGD---GKLLNALRIKNTKKNEETDLPVSGLFYAIGHTPATKIVAGQVDTDEAGY (A267)
TRR1 (219) VQGEPAPRGLVTHLKIKNVVSGVEEVVPANGLFYAVGHDPATALVKGQVETDAEGY (274)
A_1VDC (A263) VVTKPGTTQTSVPGVFAAGDVQDKKYRQAITAAGTGCMAALDAEHYLQEI (A316)
A_3ITJ (A268) IKTVPGSSLTSVPGFFAAGDVQDSKYRQAITSAGSGCMAALDAEKYLTSL (A317)
TRR1 (275) IVTKPGTSYTSVPGVFAAGDVQDRRYRQAITSAGSGCIAALEAEKYIAESEGGDEP (330)
A_1VDC (<---) (<---)
A_3ITJ (<---) (<---)
TRR1 (331) PFVATSIEQSNQGENAPPTLEYTSNPLL (358)
B
KRE2 (1) MNSVSGKYLRYILFVVLGLTILHFISSSSLPLPNANNVVSNLKPGAIKPDFSSLSQSLF (59)
A_1S4N (--->) (--->)
KRE2 (60) SEKSTEILTVLLASALALAAPAQRPAYERVNATFVTLARNEDVWDISKSIRQVEDRFNRN (119)
A_1S4N (A104) KTTMDYITPSF|--KAGKPKACYVTLVRNKELKGLLSSIKYVENKINKK (A151)
KRE2 (120) YHYDWVFINDKPFNDEFKKVTSALVSGKTHYGHIPSEHWSFPDFIDQERAAKVREDMKEK (179)
A_1S4N (A152) FPYPWVFLNDEPFTEEFKEAVTKAVSSEVKFGILPKEHWSYPEWINQTKAAEIRADAATK (A211)
KRE2 (180) QVIYGDSISYRHMCRYESGFFFRHELLNQFDYYWRVEPSVEYFCDINFDPFKFMKDNKKK (239)
A_1S4N (A212) Y-IYGGSESYRHMCRYQSGFFWRHELLEEYDWYWRVEPDIKLYCDINYDVFKWMQENEKV (A270)
KRE2 (240) YSFVLSLYEYKETVPTLWDSVKKFMEKYPQHIAEDNNMDFISSDGGDTYNLCHFWSNFEV (299)
A_1S4N (A271) YGFTVSIHEYEVTIPTLWQTSMDFIKKNPEYLDENNLMSFLSNDNGKTYNLCHFWSNFEI (A330)
KRE2 (300) GDINWLRSQEYLDYFDVLDKDGGFFYERWGDAPVHSIAAALMLKKEEVHFFNEIAYRHVP (359)
A_1S4N (A331) ANLNLWRSPAYREYFDTLDHQGGFFYERWGDAPVHSIAAALFLPKDKIHYFSDIGYHHPP (A390)
KRE2 (360) FVHCPTGEQKRLDLKCHCNPKDNFDWKGHSCTSRYFDVNKLKKPEGYEEEQD (411)
A_1S4N (A391) YDNCPLDKEVYNSNNCECDQGNDFTFQGYSCGKEYYDAQGLVKPKNWKKFRE (A442)
