# Supplementary material for: Comparative genomics allowed the identification of drug targets against human fungal pathogens
Source: BMC Genomics. 2011 Jan 27;12:75. doi: 10.1186/1471-2164-12-75 (PMC3042012; doi:10.1186/1471-2164-12-75)
Supplement: Additional file 4 — Amino acid alignment between conserved protein residues of KRE2, in the human pathogenic fungi. Amino acid sequence analysis of KRE2 protein. Af: Aspergillus fumigatus, Bd: Blastomyces dermatitidis, Ca: Candida albicans, Ci: Coccidioides immitis, Cn: Cryptococcus neoformans, Hc: Histoplasma capsulatum, Pb01: Paracoccidioides brasiliensis isolate 01, Pb3: P. brasiliensis isolate 3, Pb18: P. brasiliensis isolate 18. Positions of identity are indicated with asterisks, a semicolon indicates conserved substitutions, and a dot shows a semi-conservative substitution. [file 1471-2164-12-75-S4.PDF]

```
Kre2Pb3      GHIPSEHWSFPDFIDQERAAKVREDMKKKQVIYGDSISYRHMCRYESGFFFRHELLDQFD 210
Kre2Pb18     GHIPSEHWSFPDFIDQERAAKVREDMKKKQVIYGDSISYRHMCRYESGFFFRHELLDQFD 210
Kre2Pb01     GHIPSEHWSFPDFIDQERAAKVREDMKEKQVIYGDSISYRHMCRYESGFFFRHELLNQFD 210
Kre2Bd       GLIPTEHWSFPDFIDQQKAAKVREEMKEKQVIYGDSISYRHMCRYESGFFFRHELLNQFD 188
Kre2Hc       GVIPKEHWSFPDFIDQAKAAKVRQDMKEKQVIYGDSISYRHMCRFESGFFFRQELLQKFD 198
Kre2Ci       GKIPEEHWSFPEWIDQNKAAEARKEMEEKKVIYGGSISYRHMCRYESGFFFRHELMLNYD 224
Kre2Af       GEIPKEHWSYPDWIDQEKAKKVREEMGQKKIIYGDSESYRHMCRYESGFFFRHPLMLNYE 200
Kre2Ca       GLIPKEHWSYPPWIDQEKAALVREQMREKKIIYGHSESYRHMCRFESGFFWRQEILNDYD 230
Kre2Cn       GKIDPEHWYQPEWIDEEKATKAREEMIRKKVIYGHSVPYRNMCRFNSGFFFRHPLLANYD 200
             * *  ***  * :**: :*  .*::* .*::*** *  .**:***::****:*: :: .::

Kre2Pb3      YYWRVEPNVEFFCDINFDPFKFMKDNKKKYSFVLSLYEYKETVPTLWDSVKKFMAKYPQH 270
Kre2Pb18     YYWRVEPNVEFFCDINFDPFKFMKDNKKKYSFVLSLYEYKETVPTLWDSVKKFMAKYPQH 270
Kre2Pb01     YYWRVEPSVEYFCDINFDPFKFMKDNKKKYSFVLSLYEYKETVPTLWDSVKKFMEKYPQH 270
Kre2Bd       YYWRVEPSVEYFCDISYDPFKFMKDNKKKYSFVISLYEYKETVLTLWDSVKKFMKKYPQH 248
Kre2Hc       YYWRVEPSIEYFCDIGFDPFKFMKDNKKKYSFVISLYEYRDTVATIWDSVKKFMEKYPQH 258
Kre2Ci       YYWRVEPSVKYFCDIDFDAFKFMKDNKKKYSFVLSLHEYRETVATLWDSVKKFMEKHPEH 284
Kre2Af       YYWRVEPSIELYCDISFDPFKLMKEQNKKYSFVLSLYEYYDTIPSLWDSVKKFMGNHPEH 260
Kre2Ca       YYWRVEPDIKLYCDIDYDIFKWMKDNNKDYAFTISLPEYKETIPTLWDTTKEFIEKNPQY 290
Kre2Cn       YYWRIEPSVKFFCDLAYDPFLVMQDQNKVYGFTISLFEYIETIPTLWDAVKEFIGEHPEY 260
             ****:**.:: :**: :* *  *::::* *.*.:** ** :*: ::**:.*:*: : *::

Kre2Pb3      IAEDNNMDFISSDGGETYNLCHFWSNFEVGDINWLRSQEYLDYFDVLDKDGGFFYERWGD 330
Kre2Pb18     IAEDNNMDFISSDGGETYNLCHFWSNFEVGDINWLRSQEYLDYFDVLDKDGGFFYERWGD 330
Kre2Pb01     IAEDNNMDFISSDGGDTYNLCHFWSNFEVGDINWLRSQEYLDYFDVLDKDGGFFYERWGD 330
Kre2Bd       IAENNNLEFVSQDGGETYNLCHFWSNFEIGDLNWLRSQAYLDYFDILDKDGGFFYERWGD 308
Kre2Hc       IAEDNNLEFVSEDGGKTYNLCHFWSNFEIGDLNWLRSQAYLDYFDILDKDGGFFYERWGD 318
Kre2Ci       IVQGNNLEFVSEDGGKTYNMCHFWSNFEIGDLNWLRSKAYLDYFDVLDKDGGFFYERWGD 344
Kre2Af       IAEGNAMGFLSDDGGKTYNKCHFWSNFEIGSLEWLRSKQYLDYFDALDHDGGFFYERWGD 320
Kre2Ca       LAQNNLMDWVSDDKGQTYNGCHFWSNFEIGSLAFWRSEAYRKYFEHLDKAGGFFYERWGD 350
Kre2Cn       LPEGNAMQFVSDDGGETYNKCHFWSNFEIGDLNFWRSQPYMEFFEFLDKKGGFYYERWGD 320
             : :.* : ::*.* *.*** ********:*.: : **: * .:*: **: ***:******

Kre2Pb3      APVHSIAAALMLKKEEVHFFNEIAYRHVPFVHCPTGEQKRLDLKCHCNPKDNFDWKGHSC 390
Kre2Pb18     APVHSIAAALMLKKEEVHFFNEIAYRHVPFVHCPTGEQKRLDLKCHCNPKDNFDWKGHSC 390
Kre2Pb01     APVHSIAAALMLKKEEVHFFNEIAYRHVPFVHCPTGEQKRLDLKCHCNPKDNFDWKGHSC 390
Kre2Bd       APVHSIAAALMLKKEEVHFFNEIAYYHVPFYHCPTGEQTRLDLKCHCNPKNNFDWKGYSC 368
Kre2Hc       APVHSIAAALMLKKEEIHFFNEIAYRHVPFVHCPTGEQMRLDLKCHCNPRDNFDWKGYSC 378
Kre2Ci       APVHSIAASILLKKEEVHFFDQIGYYHVPFTHCPTGEQRRTEWKCACNPGDNFDWKGHSC 404
Kre2Af       APVHSIAAGIMLKKEEIHFFNEIAYYHIPFTHCPTSEQMRLDLKCHCNPSENFDWKGYSC 380
Kre2Ca       APVHSIAAALFLPREKIHFFEDVGYYHVPFTNCPVDKEVRKARNCNCDPNKDFTWRGYSC 410
Kre2Cn       APVHSIGAALFAKKEQIHFFDDIGYRHEPFQHCPQGD-AHTRGNCWCDQANNFDWEWYSC 379
             ******.*.::  :*::***::.* * ** :** ..  :   :* *:  .:* *. :**
```
